# Welding Characteristics Analysis and Application on Spacecraft of Friction Stir Welded 2A14-T6 Aluminum Alloy

**DOI:** 10.3390/ma12030480

**Published:** 2019-02-04

**Authors:** Haitao Luo, Tingke Wu, Jia Fu, Wei Wang, Ning Chen, Haonan Wang

**Affiliations:** 1 Shenyang Institute of Automation, Chinese Academy of Sciences, Shenyang 110016, China; wutingke@sia.cn (T.W.); fujia@sia.cn (J.F.); WangWei@stumail.neu.edu.cn (W.W.); chenning2017@126.com (N.C.); 1770174@stu.neu.edu.cn (H.W.); 2 Institutes for Robotics and Intelligent Manufacturing, Chinese Academy of Sciences, Shenyang 110016, China; 3 School of Mechanical Engineering, Shenyang Ligong University, Shenyang 110159, China; 4 Institute of Mechanical Engineering and Automation, Northeastern University, Shenyang 110819, China

**Keywords:** friction stir welding (FSW), finite element model (FEM), microstructure, mechanical properties, aerospace application

## Abstract

According to the actual size parameters, the finite element model (FEM) of friction stir welding (FSW) was established, and the FEM was updated by experiments. The FSW of the 2A14-T6 high-strength aluminum alloy was simulated under a reasonable welding process parameter range, and the welding process parameters with good simulation effect were determined. The test was carried out under the same parameters, and the axial force of the FSW tool and temperature of the workpiece measuring point were collected. The comparison between the simulated data and the experimental data is reasonable, indicating the correctness of the FEM. The microstructure analysis of the welded joint shows that the grain size in the upper part of the weld nugget was smaller than that in the middle and lower parts, and there are obvious boundaries of grain size in each region of the joint. The hardness of the joint in the upper layer is higher than that in the middle and lower layers, and the minimum Vickers hardness value of the joint appears near the interface between the thermo-mechanically affected zone and the heat-affected zone on both sides of the weld. Tensile testing shows that the strength coefficient of the joint reaches 82.5% under this process parameter, and the sample breaks at the intersection of the material flow during stretching. After analyzing the final mechanical properties of the joint, we found that a degree of aerospace application can be achieved. Under this parameter, the welding test was carried out on the top cover of the rocket fuel tank. Firstly, melon valve welding, which is relatively difficult in welding conditions, was carried out, and a high-quality joint with good surface and no defects was obtained.

## 1. Introduction

Aluminum alloys are widely used in electrical appliances, ships, aerospace, transportation, and other industries. Aluminum alloys have the advantages of high specific strength, low density, corrosion resistance, good toughness, and good molding process, and are widely used in the high-end equipment manufacturing industry. As an important metal connection technology, welding is an indispensable processing technology in the production of structural parts. However, due to the characteristics of high thermal conductivity, low melting point, and high thermal expansion rate in the welding process of aluminum alloy, the traditional fusion welding process is prone to defects such as welding hot cracks, inclusions, porosity, alloy element burning, and welding residual deformation, which lead to a decrease in weld quality. Compared with fusion welding, friction stir welding has the advantages of solid state connection, lower production cost, and excellent joint performance in aluminum alloy welding [1,2,3,4]. Friction stir processing (FSP) is a solid-state surface modification process, which causes severe plastic deformation, mixing of material, and lower temperature exposure, leading to significant microstructural refinement, densification, and homogeneity of the processed layer. Friction stir welding (FSW) uses high-speed rotating welding tools to heat the workpiece to make the material flow in a thermoplastic manner and realize weld forming under the forging action of the shaft shoulder [5,6,7,8].

As a new green welding technology, 2A14-T6 aluminum alloy belongs to the Al‒Cu‒Mg‒Si series aluminum alloy and has a high specific strength, specific modulus, fatigue strength, fracture toughness, and corrosion resistance stability. It has been identified as a new generation of aerospace structural material at home and abroad and has been widely studied. Due to the harsh application environment, the requirements for weld quality are particularly strict. Therefore, the weld quality and connection performance directly affect the safety and service life of the structural parts and the entire piece of equipment. In view of its important space application background, it is of great significance for the development of the space manufacturing industry to study it in depth.

The friction stir welding process can be visually studied through experiments. The research mainly includes precipitation evolution and microstructure‒property correlation [9]. The influence of welding parameters on the properties and microstructure of 2A14-T6 aluminum alloy joints has not been reported in many studies, but is very important from the perspective of practical application. Su [10] measured lateral force, axial force, and tool torque simultaneously by monitoring the output torque of servo motor and spindle, and checking the factors affecting welding quality. Under different combinations of tool speed and welding speed, the measurement results laid a foundation for process optimization. Liu [11] studied the friction stir welding process of 2219-T6 aluminum alloy. The results showed that a void defect is formed in the joint when the rotation or welding speed is quite high. As the rotation speed or welding speed increases, the tensile strength of the joint first increases to a maximum value and then sharply decreases due to the occurrence of a void defect. Shojaeefard et al. [12] focused on the microstructural and mechanical properties of the friction stir welding of AA7075-O to AA5083-O aluminum alloys. Weld microstructures, hardness, and tensile properties were evaluated in as-welded condition. Tensile tests indicated that the mechanical properties of the joint were better than in the base metals.

In addition to experimental study, numerical simulation is also considered to be an important tool to study the effect of the welding process on weld quality. Narges Dialami [13] studied the metallurgical and microstructure of friction stir welding (FSW) from the aspects of grain size and microhardness. The strain rate and temperature histories obtained from the numerical model are stored on the tracers. The relationship among the grain size, microhardness, strain rate, and temperature is obtained using the Zener‒Hollomon parameter and Hall‒Petch relationship. Jamshidi [14] simulated the temperature field of friction stir welding by ABAQUS software. The computing time is saved by non-uniform mesh. The results show that the temperature field is asymmetrical in relation to the center of the weld, and the temperature of the advance side of the weld is slightly higher than that of the retreat side of the weld. The welding process of friction stir welding can be divided into three stages: the plunging stage, the dwelling stage, and the welding stage. The heat produced by the interaction between the shaft shoulder and the workpiece interface is friction heat when calculating the temperature field. The heat production of the agitated needle is mainly caused by the shear stress [15,16,17]. The preheating process helps the material reach the plastic state as soon as possible and reduces welding defects [18,19].

Due to the uncertainty of parameter interval setting in the test process, the difficulty and workload of the test are greatly increased. In 2A14-T6 aluminum alloy friction stir welding, the influence of welding speed, rotating speed, and pressing down on the welding quality was analyzed by changing the welding process parameters. When the welding process parameters are appropriate, a welding joint with beautiful shape, smooth and compact appearance, no internal defects, and excellent quality can be obtained [20,21,22]. In this paper, the finite element theory of friction stir welding is analyzed, and the FEM is modified by the test data. The modified FEM is used to simulate the full working conditions of FSW. It can effectively determine the process parameters of better welding quality and has a guiding significance in the real welding process.

To sum up, the weld quality is related to factors such as spindle speed, welding speed, welding inclination, etc. Therefore, in order to study a better-quality friction stir welding process, it is necessary to analyze and verify the process parameters. The method of numerical simulation can be used to carry out the verification work in the early stage, which can provide an effective reference for the later test stages. The microstructure and mechanical properties of the joint were analyzed. The welding quality was determined by considering the strength and plasticity of the welded joint on the basis of a large number of process experiments. Welding process parameters with good quality were applied to the roof welding of rocket fuel tank.

## 2. Finite Element Model of FSW

### 2.1. Model Description

**Figure 1:**
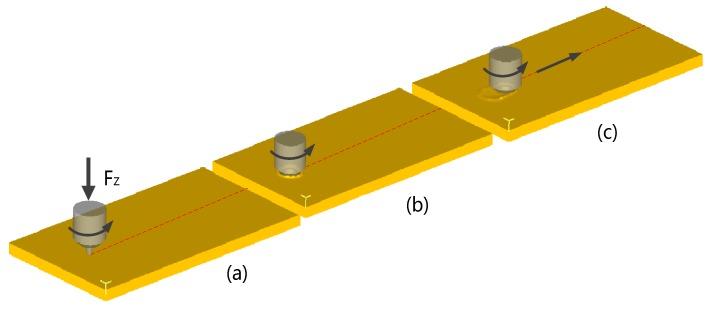
Schematic of the friction stir welding (FSW) process: (**a**) Plunging, (**b**) dwelling, (**c**) welding.

The friction stir welding process can be roughly divided into the following stages: the plunging stage, the dwelling stage, and the welding stage. The process is shown in Figure 1. In the first two stages of welding, the tool and the workpiece to be welded generate heat by friction, the thin layer of material around the FSW tool is softened into a viscous flow state, the viscous fluid material is plastically deformed under the stirring of the FSW tool, the plastic deformation process is accompanied by deformation and heat generation, and the temperature around the FSW tool is continuously increased. When the frictional heat generation and the plastic deformation heat generation enter a stable state, the thin layer of the viscous fluid material around the FSW tool reaches a stable thickness and the welding enters a stable state. In the stable welding stage, due to the rotation and movement of the FSW tool, the front edge viscous fluid material fills the cavity created in the rear of the welding direction, forming a tight weld under the action of forging force [23,24].

The FSW finite element simulation is the same as the real welding process and is divided into three stages: plunging, dwelling, and welding. During the pressing process, the FSW tool is inclined to the trailing edge by 2.5°; the tool travels at a defined rotational speed with a feed velocity of 6 mm/min, and the shoulder maintains a plunge depth of 0.25 mm. After the pressing reaches the specified depth, it stays for 10 s. Finally, the FSW tool is welded at a speed of 80 mm/min.

### 2.2. Geometric Model

**Figure 2:**
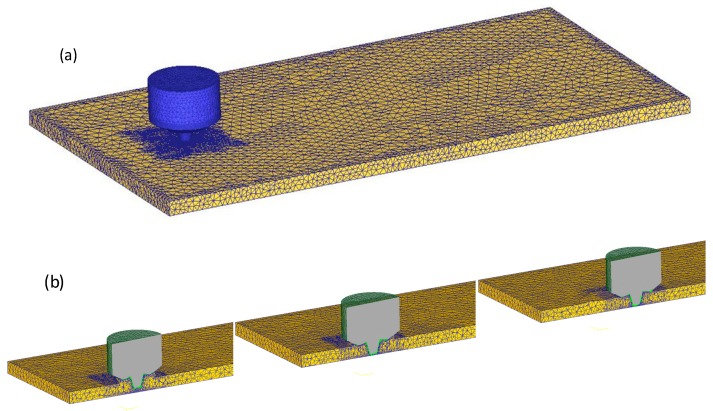
Mesh model of tool and workpieces used in the simulation process: (**a**) Meshing of tool and workpiece; (**b**) the welding simulation process and mesh window move with the welding feed.

The FSW process is a dynamic nonlinear analysis based on the Lagrange method. On the basis of previous process experiments, the welding parameter interval was roughly determined by considering the strength and plasticity of the welded joint. The whole process of friction stir welding of a 2A14 aluminum alloy was numerically simulated by finite element software Deform-3D. The material of the tool was W6, and the workpiece was 2A14-T6 lightweight aluminum alloy of 150 mm × 100 mm × 6 mm size. The workpiece was divided into 15,256 nodes and connected to form 65,450 tetrahedral elements. Tool steel W6 was connected to 9584 nodes to form 43,268 tetrahedral elements. Tool and workpiece meshing are shown in Figure 2a. The yield strength of the workpiece was much less than the strength of the material used in the tool, so the tool was defined as a rigid body that could conduct heat conduction in the numerical simulation process. The diameter of the shoulder of the tool was 16.3 mm, the maximum use diameter of the tool pin was 8.15 mm, and the length of the tool pin was 5.6 mm. The specific dimensions are shown in Table 1.

In the process of numerical simulation, the powerful adaptive mesh re-division technique of the software is used to solve the problem of mesh distortion caused by material flow in the welding process. The mesh of the contact area is finely divided through the mesh window to obtain more accurate results, and the refined mesh window moves along with the mixing head during welding feed. The welding simulation process and mesh window move with the welding feed as shown in Figure 2b. The boundary condition set by the FSW model is that the workpiece is constrained by setting zero speed in three directions, thus ensuring that all degrees of freedom of the workpiece are limited [25,26,27]. The movement of the FSW tool is defined by the rotation in the Z direction and the feeding movement in the X direction. The convection heat coefficient between the workpiece and the environment is set to 20 W/m^2^/°C [28], and the convection heat coefficient of the workpiece bottom surface is set to 200 W/m^2^/°C to consider the thermal conduction effect of the backing plate [28].

### 2.3. Finite Element Formulation

In the finite element simulation of plastic forming, according to the nonlinear constitutive of materials, the finite element method is divided into three categories: elastic–plastic, rigid plastic, and viscoplastic. These methods use the energy equation to establish a functional analysis and perform variational solving. In friction stir welding, the elastic deformation of the component material is negligible compared to the plastic deformation. Considering that friction stir welding is formed at high temperatures, the material exhibits a viscous property, so the component is set as an incompressible rigid viscoplastic model, and a rigid viscoplastic finite element method is used for finite element analysis. A rigid viscoplastic model with von Mises yield criteria is used [28,29,30]. The stress‒strain rate relationship is used in the deformation zone, and the formulas are as shown in Equations (1)–(3):

(1) $${\dot{\varepsilon }}_{ij}=\frac{3}{2}\frac{\dot{\bar{\varepsilon }}}{\bar{\sigma }}\sigma_{ij}^{\prime }$$

(2) $$\bar{\sigma }=\sqrt{\frac{3}{2}}\sigma_{ij}^{\prime }$$

(3) $$\dot{\bar{\varepsilon }}=\sqrt{\frac{3}{2}{\dot{\varepsilon }}_{ij}{\dot{\varepsilon }}_{ij}}$$

where $\bar{\sigma }$, $\dot{\bar{\varepsilon }}$ , ${\dot{\varepsilon }}_{ij}$, $\sigma_{ij}^{\prime }$ are effective stress, effective strain rate, strain rate component, and deviatoric stress component, respectively. The finite element formula of rigid viscoplastic materials is based on the variational principle, in which the allowable speed should meet the conditions of compatibility and incompressibility, as shown in Equation (4).

(4) $$\pi =\int_{V}E({\dot{\varepsilon }}_{ij})dV-\int_{S_{F}}F_{i}u_{i}dS$$

where $F_{i}$, V, $S_{F}$ and $E({\dot{\varepsilon }}_{ij})$ are functions of surface traction, workpiece volume, stress surface, and plastic deformation power, respectively. The penalty function of incompressibility is added to eliminate the incompressibility constraint on the allowable velocity field. The actual velocity field can be determined by the stable value of the change equation [31], which is expressed as:

(5) $$\delta \pi =\int_{V}\bar{\sigma }\delta \dot{\bar{\varepsilon }}dV+\lambda \int_{V}{\dot{\varepsilon }}_{V}\delta {\dot{\varepsilon }}_{V}dV-\int_{S_{F}}F_{i}\delta u_{i}dS$$

Equation (5) is the basic equation of the Lagrange finite element method, where ${\dot{\varepsilon }}_{V}$ is the volume strain rate, $\delta \dot{\bar{\varepsilon }}$ and $\delta {\dot{\bar{\varepsilon }}}_{V}$ are the strain rate changes derived from $\delta u_{i}$, and *λ* is the penalty factor for the volume incompressible condition. To ensure an incompressible condition, the *λ* value is an infinite positive number.

### 2.4. Material Model

The proper selection of a material model is very important for the accurate calculation in the simulation process. The material changes from a solid state to a viscous state, and the accuracy can be improved by defining a wide range of strains. The stress‒strain curves at different temperatures in the material library are used as input to the simulation, and the isotropic strengthening principle is considered. Flow stress is defined as a function of strain, strain rate, and temperature, as shown in Equation (6).

(6) $$\bar{\sigma }=\bar{\sigma }(\varepsilon ,\dot{\bar{\varepsilon }},T)$$

where $\bar{\sigma }$ is the flow stress, ε is the strain, $\dot{\bar{\varepsilon }}$ is the strain rate, and *T* is the temperature.

### 2.5. Thermal Model

Friction stir welding is a thermo-mechanical coupling process, and the temperature distribution is determined by the heat conduction equation of Fourier’s law, taking into account the transient heat transfer factors, such as Equation (7).

(7) $$k\Delta^{2}T+\dot{q}=\rho c_{p}\frac{\partial T}{\partial t}$$

where *k* is the thermal conductivity of the material, *T* is the temperature, *ρ* is the density of the material, and $c_{p}$ is the heat capacity per unit mass of the material.

(8) $$\dot{q}={\dot{q}}_{f}+{\dot{q}}_{p}$$

where the heat generated in the process is the frictional heat generation, which is the heat generated by the plastic deformation of the material.

(9) $${\dot{q}}_{p}=\eta \bar{\sigma }\times \dot{\varepsilon }$$

where *η* is the inelastic heat fraction, which is defined as the amount of mechanical energy converted to heat energy and it is taken to be 0.9 [32]; the fraction of the remaining energy is utilized in changing dis-location density, sub-grain boundaries, phase transformation and evolution, etc.

### 2.6. Friction Model

The contact process between the tool and the workpiece in FSW is complicated. Due to the lack of experimental data, researchers assume that the contact conditions are different. Some researchers use coulomb friction to assume the process [33]. Other scholars believe that the tool and workpiece are in a state of adhesion [34,35]. This consideration is used because the yield strength of the material is the limiting condition of the contact stress.

(10) $$\bar{\tau }=m\tau_{\max}$$

where $\bar{\tau }$ is the contact stress at the interface between the tool and the workpiece, *m* is the shear coefficient, and $\tau_{\max}$ is the shear yield strength of the material, which is 0.577 times the von-Mieses yield criterion.

**Figure 3:**
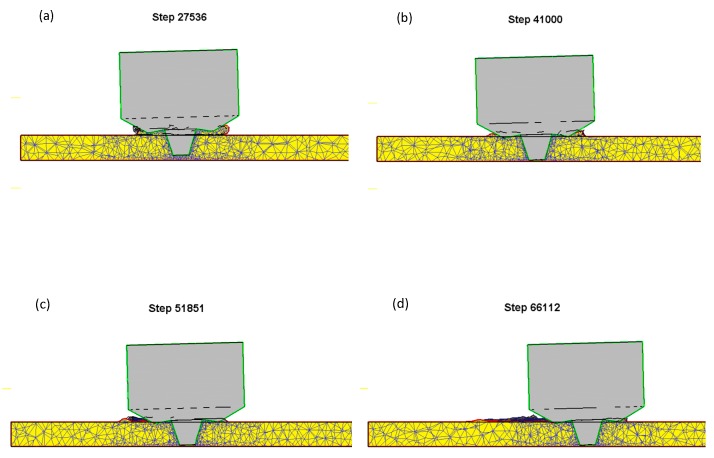
Simulation results of welding process: (**a**) Plunging phase, 43 s; (**b**) dwelling phase, 64 s; (**c**) welding phase, 79 s; (**d**) welding phase, 98 s.

Several sets of parameter simulations were simulated, and the cross section was intercepted along the feed direction of welding to observe the existence of weld defects. From the cross section, it can be seen that there was material overflow in the pressing stage, and the welding result is shown in Figure 3. The image shows the outer contour of the mixing head in green, and the contour of the workpiece and the deposited material in red. If there were any defects such as air holes and cracks, they would be shown in red. From the simulation results, it can be seen that 2A14-T6 aluminum alloy had a good effect in the process of plunging, dwelling, and welding under this process parameter; no welding defects were found, and a good-quality weld seam was obtained. The test could verify the welding parameters relatively accurately.

## 3. Experimental Methods and Data Acquisition

Friction stir welding is essentially a welding method using friction heat as the welding heat source, so it is an effective way to evaluate the quality of joints by using heat input. The heat input of friction stir welding can be expressed as:

(11) $$q_{k}=k^{\prime }n/v$$

where $k^{\prime }$ is a constant, *n* is the rotation speed of the tool, and v is the welding speed. It can be seen that the parameter n/v directly represents the size of the welding heat input. For a given FSW tool structure and plunge depth, the quality of the friction stir welded joint is primarily dependent on the heat input factor. Therefore, the adjustment of the process parameters can effectively control the weld microstructure of the heat-affected zone and improve the performance of the welded joint.

The base metal for this investigation was a 6-mm-thick 2A14-T6 aluminum alloy plate with dimensions of 300 mm long by 150 mm wide. The chemical composition and mechanical properties of the base metal are listed in Table 2 and Table 3 [36]. The original 2A14-T6 aluminum alloy base material has a coarse strip grain structure, and massive second phases are uniformly distributed in grain boundaries and grains. When the second phases are uniformly distributed in the matrix phase as fine dispersed particles, a significant strengthening effect occurs, which is called second phase strengthening. The atomic percentages of Al, Cu, Mg, and Si, the main elements of 2A14-T6 aluminum alloy base metal, are 66.22%, 32.23%, 1.08%, and 0.47%, respectively [37]. The massive second phase is essentially an aggregate of precipitated phase Al2Cu, which has a certain strengthening effect. The main reason for the strengthening of the second phase is the interaction between them and dislocation; which hinders dislocation movement and improves the deformation resistance of aluminum alloy.

The FSW tool used for welding was a shoulder concave structure; the tool pin was a conical thread structure, the diameter of the shoulder of tool was 16.3 mm, the cone angle of the tool pin was 15°, the length of the tool pin was 5.6 mm, and the overall size was consistent with the parameters of the simulation model. The welding process was completed on a self-developed high-precision friction stir welding robot. During the welding process, the spindle speed was 800 r/min, the welding speed was 80 mm/min, the plunge depth of the tool shoulder was 0.25 mm, and the tool was titled by 2.5° towards its trailing edge. The overall welding parameters were consistent with the simulation parameters.

**Figure 4:**
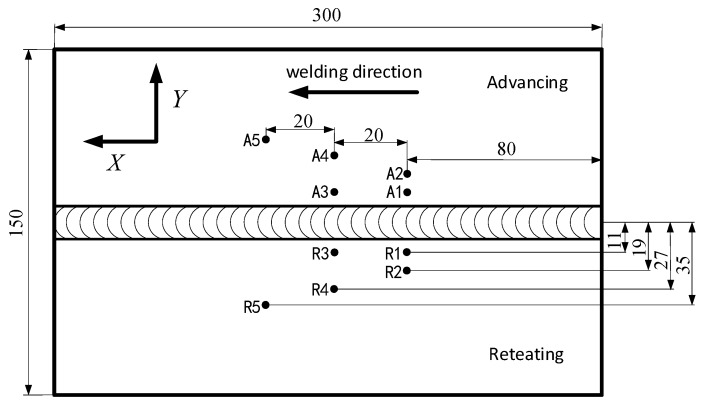
Location distribution of temperature measuring points.

The FEM numerically simulates the temperature field on the workpiece, so the temperature measurement on the workpiece can be compared with the finite element simulation data, and the FEM can be modified to ensure the correctness of the FEM simulation. During the welding process, K-type sheathed thermocouples were used to synchronously collect the temperature of the measuring points, and the first set of measuring points was set at 80 mm from the initial welding position, because the temperature near the initial position did not reach a steady state during the welding process, while the temperature also reached a relatively steady state value with the stable welding seam formation. Specific temperature measurement points are shown in Figure 4.

**Figure 5:**
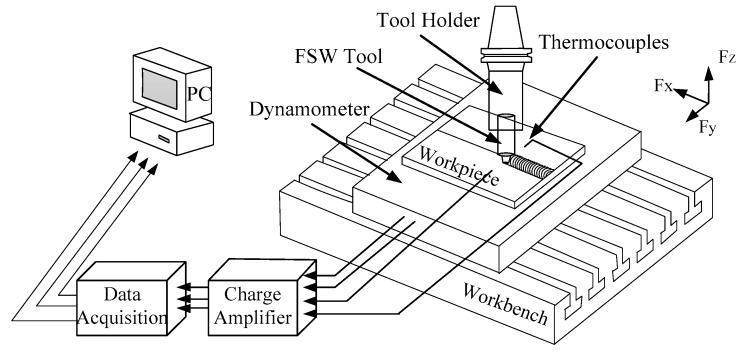
Simplified diagram of FSW temperature and force measurement.

The welding materials and dimensions used in the test was a 2A14-T6 aluminum alloy plate, which was the same as the simulation, and a ME-K6D175 pressure sensor installed under the workpiece. The mechanical sensor can synchronously output force and torque in the XYZ direction with high acquisition frequency and accuracy. The temperature measurement and force measurement diagrams are shown in Figure 5. The acetone reagent was used to remove the oil stain from the surface of the workpiece, and then the workpiece was clamped along the length of the workpiece on the pressure sensor and welded. The welding parameters were the same as the simulation parameters. The axial force and temperature data were synchronously collected during welding.

After the welding was finished, the cross section of the joint was taken perpendicular to the welding direction. After rough grinding, fine grinding, and polishing, the sample was corroded with a mixed acid solution (3 mL HNO_3_ + 6 mL HCL + 6 mL HF + 150 mL distilled water), and the internal shape of the joint and the microstructure of each area were analyzed with an optical microscope.

On the cross section of the polished specimen, Vickers hardness tests were carried out on the upper, middle, and lower layers of the joint by a microhardness tester (Beijing TIME High Technology Ltd, Beijing, China). The test position was 1, 3, and 5 mm from the upper surface of the weld. The joint was cut into standard specimens by a wire electric discharge machine, the normal temperature tensile test was carried out on a mechanical property tester, the average value of the three specimens was used as the evaluation result of the joint performance, and then the tensile fracture characteristics of the joint were analyzed.

## 4. Results

### 4.1. Comparison and Verification between Test and Simulation

**Figure 6:**
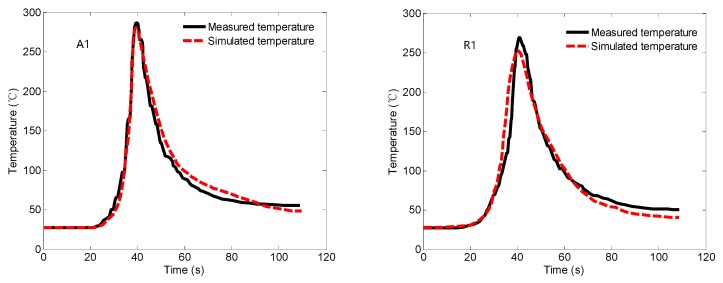
Comparison between simulated and measured temperature.

Figure 6 shows the temperature change curve of each measuring point in the FSW process. A1 is the temperature measurement point on the advancing side of the workpiece; R1 is the temperature measurement point on the retreating side of the workpiece, which is 11 mm from the center of the weld. It can be seen that, although the simulation result of the temperature of the measuring point is different from the measured result, with the actual result faster than the simulation result in the cooling stage, the overall trend of the curve is similar. Comparing the temperature curves of A1 and R1, it can be found that the temperature on the advancing side is higher than the temperature on the retreating side at the symmetrical position, and the experimental and simulated temperature curves of other measuring points have similar corresponding relationships with A1 and R1. Generally, the simulation result of the temperature measurement point is in good agreement with the measured result.

**Figure 7:**
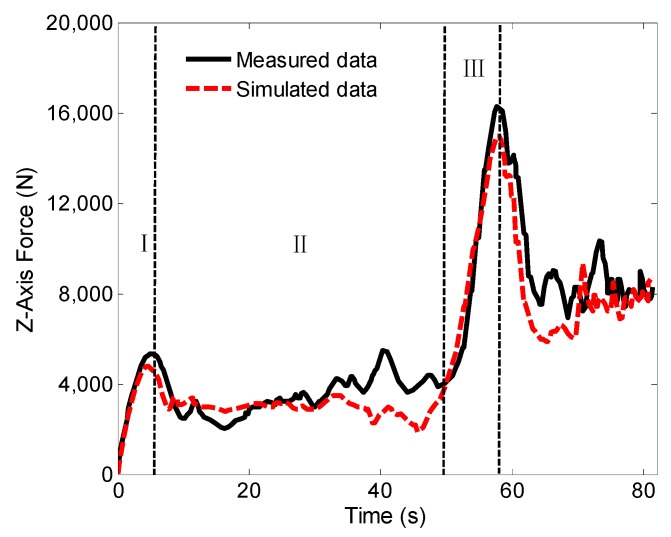
Axis force curve in Z direction.

During FSW welding, the axial force of the Z axis was tested. Figure 7 shows the comparison between the test results and the simulation results. It can be seen from the curve that the temperature gradually rises and reaches a peak in the first stage of the curve, which is equivalent to the contact between the stirring pin and the workpiece. The temperature increases to soften the base material around the tool pin and promote the pressing down of the tool. In the second stage, the axial force is reduced, the material around the tool pin is gradually softened, and the mechanical interaction between the tool and workpiece allows the FSW tool to continue to be pressed into the workpiece without increasing the external force. In the third stage, when the tool shoulder comes into contact with the extruded material, the axial force begins to increase gradually. After the shoulder is pressed down to a certain amount, the extruded material at the outer ring edge of the shoulder forms a flash, which is also fully reflected in the simulation process. However, the overall axial force output by simulation is slightly lower than the actual test results, but the overall trend is in good agreement. In summary, the comparison of temperature curve and axial force curve shows that the simulation results are accurate, and the FEM is reliable.

### 4.2. Weld Forming Characteristics and Microstructure

**Figure 8:**
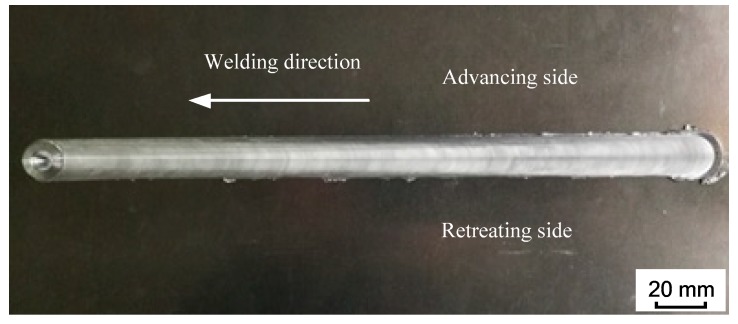
Welding effect diagram.

Figure 8 shows the forming of the weld surface. From the welding results, it can be seen that the weld surface is smooth and compact, and the arc pattern is uniform and compact. This shows that the welding effect is good under this process parameter, with only a few flash defects, and the weld has good forming characteristics.

**Figure 9:**
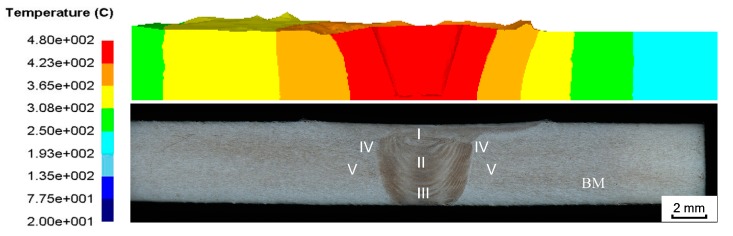
Cross-sectional comparison of joints. BM is base material.

Figure 9 shows a comparison of the simulated temperature field in the cross section of the joint and the metallographic structure of the joint. The observation shows that the shoulder affected zone (SAZ, zone I) in the cross section of the joint is connected to the weld nugget zone (WNZ, zone II), the thermo-mechanically affected zone (TMAZ, zone IV) is adjacent to the above two zones, and the heat-affected zone (HAZ, zone V) is adjacent to the TMAZ. In the FEM, the temperature in the contact area with the tool is relatively high and gradually decreases to both sides.

**Figure 10:**
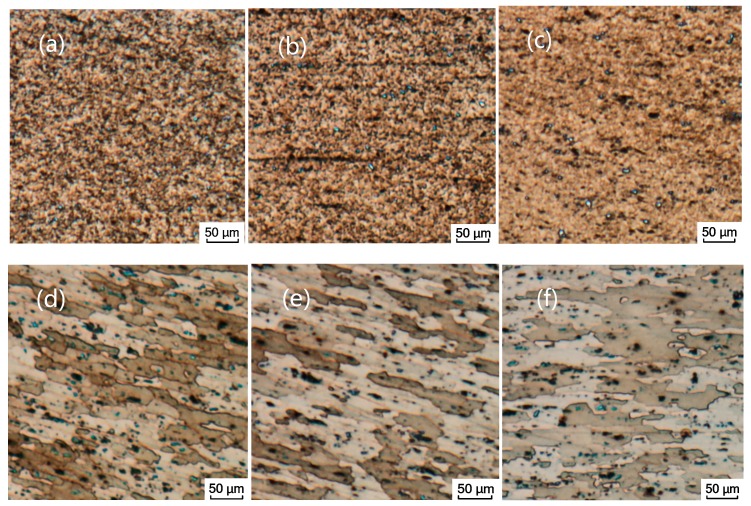
Microstructure of different regions: (**a**) Upper layer of the weld nugget zone (WNZ); (**b**) middle layer of the WNZ; (**c**) lower layer of the WNZ; (**d**) thermo-mechanically affected zone (TMAZ); (**e**) heat-affected zone (HAZ); (**f**) base metal (BM).

The comparison shows that the temperature gradient distribution has a correlation with the change of metallographic structure, the temperature in the contact area with the tool is the highest, and the high-temperature coverage area is generally distributed in the SAZ and WNZ regions. The material in this area is stirred by the rotation of the FSW tool, the material reaches a viscous state, and the grain structure is refined. The material on both sides of the viscous flow layer is in a thermoplastic state and has a certain rheological resistance. The material does not have plastic flow, but the grain size of the material in this area is bent, elongated, and grown, and finally the TMAZ and HAZ regions of the joint cross section are formed. Figure 10 shows the microstructure distribution of each region [36].

### 4.3. Joint Performance Test

**Figure 11:**
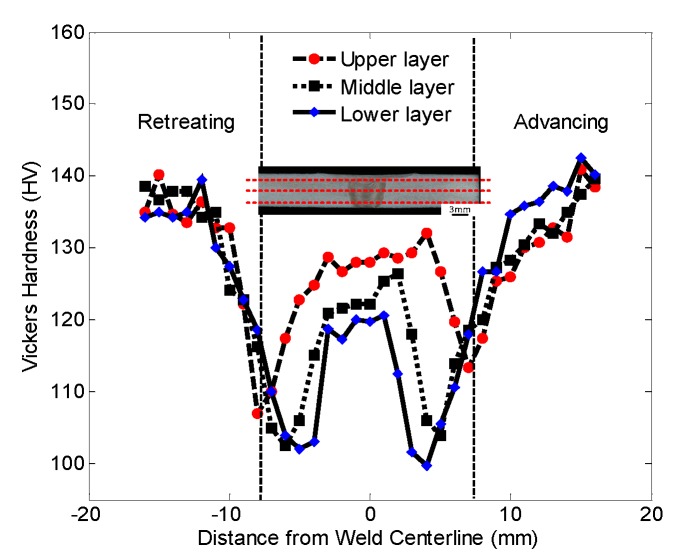
Vickers hardness distribution of joint.

The Vickers hardness test was carried out on the cross section of the welded joint. The test results are shown in Figure 11. It can be seen that the joint hardness exhibits a “W”-shaped distribution. The curve marks the position of the outer contour of the shoulder with a vertical dashed line. The Vickers hardness of the weld TMAZ, HAZ, and WNZ areas is lower than that of the base metal. The minimum Vickers hardness value of the joint appears in the vicinity of the TMAZ and HAZ interfaces on both sides of the weld.

Table 4 gives the joint tensile test results. It can be seen that the performance test results of the three tensile specimens are similar. Under the selected process parameters, the tensile strength of the joint is 379.32 MPa, which is 82.5% of the strength of the base metal, but the plastic deformation ability of the joint during stretching is weak, and the elongation after fracture is only 58.5% of the base metal.

**Figure 12:**
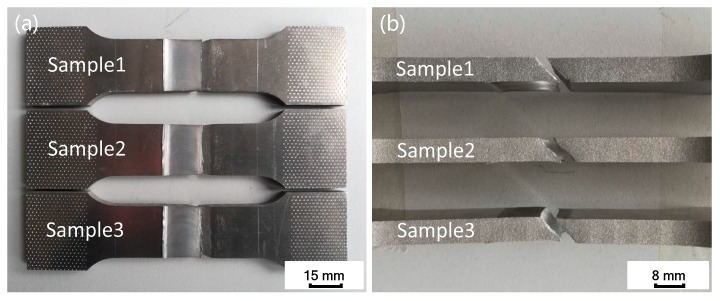
Tensile fracture locations of joint: (**a**) Tensile specimen front face; (**b**) tensile specimen side face.

Figure 12 shows the fracture position of tensile specimens, where the left side of each tensile specimen is the weld advancing side and the right side is the weld retreating side. It can be seen that all three tensile specimens are fractured on the retreating side of the weld, and the inclined fracture surface is at an angle of nearly 45° with the tensile direction. The strength of the material flow intersection area on the backward side of the joint is relatively weak, where microcracks tend to initiate and propagate during stretching, and eventually lead to fracture of the joint.

## 5. Discussion and Application

**Figure 13:**
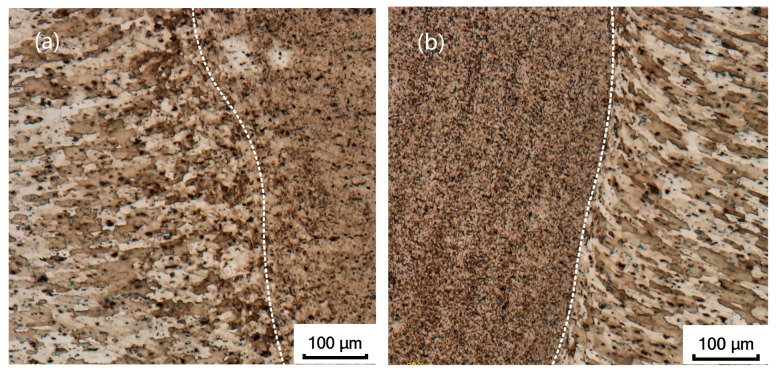
Boundary between WNZ and TMAZ: (**a**) Retreating side; (**b**) advancing side.

By analyzing the microstructure of the joint, it can be found that friction stir processing can overcome the difficulties encountered in the fusion route of surface modification like cracks, porosity, segregation, and grain growth. There are obvious boundaries between the WNZ, TMAZ, HAZ, and the base material (BM), and the boundary between the WNZ and the TMAZ is particularly obvious, as shown in Figure 13. The microstructure of each zone is different. The microstructure of the weld nugget zone is relatively small, and the microstructure of the heat-affected zone is slightly larger than that of the weld nugget zone. The microstructure of the heat-affected zone is affected by the rotation of the FSW tool and the heat dissipation of metal rheology during welding, and the grain size grows and coarsens. Compared with the base material structure, the rolling characteristics of the heat-affected zone structure are reduced, and the coarsened grain size is close to the base material [38,39].

The frictional heat generated by the rapid rotation of the FSW tool promotes dynamic recrystallization of the weld metal to refine the grain, so that the weld has a finer microstructure than the BM. Therefore, the performance improvement of friction stir welding joints is mainly achieved through the fine grain strengthening (FGS) of the weld microstructure.

The mechanical properties of the welded joints with the above process parameters were analyzed. Due to the difference of heat input in the upper, middle, and lower parts of the weld, the microstructure of the weld closer to the upper part was refined significantly, and the hardness of the upper part was significantly higher than that of the lower part. The hardness curve shows that the hardness of the advancing side was slightly higher than that of the retreating side.

In this paper, the feasibility of obtaining process parameters by means of simulation was discussed first. Considering the large workload and high testing cost of optimizing process parameters by means of experiments alone, if the simulation and experiments have a high matching degree, the finite element model can be reused in the subsequent optimization of process parameters and applied to the preliminary analysis in other welding fields. The finite element model was updated through experimental data collection to improve the simulation accuracy. Considering the cost and rigor, the welding test was carried out on a flat plate first, and the microstructure and mechanical properties of the flat plate were analyzed after the welding test. It was found that it could reach the space application level. Finally, the process parameters were applied to the curved surface welding of the rocket top cover by the friction stir welding robot developed independently.

**Figure 14:**
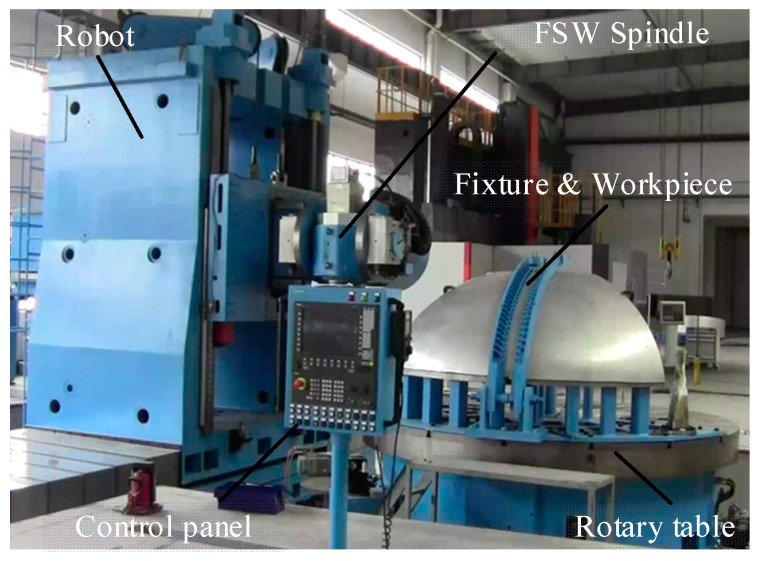
Friction stir welding robot.

Most of the existing friction stir welding equipment is based on the transformation of numerical control machine tools, which can only be adapted to the connection of regular welds, have a single function, and lack process flexibility. It is difficult to realize the welding of complex weld workpieces. In view of the large thin-walled curved surfaces that need to be welded in the aviation and aerospace fields, the number of welds is large and presents complex curves in three-dimensional space [40]. The friction stir welding robot developed for aerospace needs is shown in Figure 14. It mainly applies to the welding of any complex curved surface in three-dimensional space, such as large rocket launchers, space station segments, or aircraft external skin structures in the fields of aerospace and national defense.

**Figure 15:**
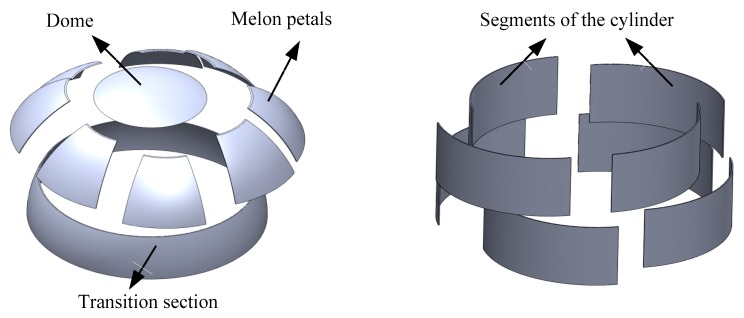
Structural composition of rocket roof and barrel.

Taking the skin structure of the rocket as an example, the skin is made of 2A14-T6 aluminum alloy. The previous research on welding process parameters can be applied to the welding of the structure. The welding of the rocket skin is mainly divided into two working conditions, namely the welding of the rocket dome and the welding of the cylinder. The structural composition of the rocket top cover and the cylinder is shown in Figure 15. There are five main kinds of welding processes applied to them: melon valve welding, melon top ring seam welding, melon bottom ring seam welding, cylindrical ring seam welding, and cylindrical longitudinal seam welding. The melon flap welding is mainly the welding between the melon flaps of spherical or ellipsoidal components. The welding trajectory is from the bottom to the top of the FSW tool axis. The axial direction of the mixing head is the normal direction of the curved surface of the workpiece to be welded.

**Figure 16:**
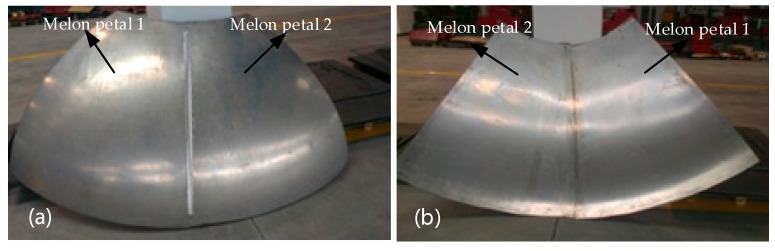
Melon petal welding of rocket roof: (**a**) Front welding effect of melon petal welding; (**b**) back welding effect of melon petal welding.

The welding of the 2A14-T6 aluminum alloy rocket roof was carried out using a large-scale stirring welding robot developed independently. As melon flap welding is relatively difficult in the welding conditions, the welding between the two melon flaps of the roof was carried out first, and the welding process proceeded smoothly. Figure 16 shows the welding results of the front and back sides of the melon flap welding. It can be seen that the surface of the curved weld is smooth and flat, the weld formation is uniform and compact, and there are almost no flash defects, while the back side of the weld is relatively tight, no weld defects are found, and the weld has good forming characteristics. It can be concluded that the above process can be applied well to the welding of large curved 2A14-T6 aluminum alloy, and the welding quality is ideal.

## 6. Conclusions

According to the actual size parameters, a friction stir welding FEM was established, and the FEM was modified through experiments. The friction stir welding numerical simulation of 2A14-T6 high-strength aluminum alloy was carried out in a reasonable process parameter range, and the welding process parameters with good simulation effect were determined. The simulation process parameters are verified by experiments, which can effectively reduce the blindness of the experiment, and are beneficial to the determination of process parameters and the economy. The finite element simulation model of friction stir welding was established to solve the temperature, axial force, and other data. The numerical simulation results showed that the temperature was the highest in the weld nugget region, and the temperature cloud picture presented a V shape. FSP is a solid surface modification process, and the material undergoes severe plastic deformation. The mixing and extrusion of materials resulted in significant microstructure refinement of the welding area.

- Mechanical and temperature data acquisition experiments of the simulated process parameters were carried out, and the simulated data were compared with the experimental data. The curves correspond well, which verifies the reliability of the FEM.
- The microscopic analysis of the joint shows that the friction stir process denatured the metal on the aluminum alloy surface. The microstructure of the weld nugget area was refined evenly, and it was obviously refined from the TMAZ to the HAZ, in which the material grains in TMAZ and HAZ were bent, elongated, and grown.
- The tensile test of the joint shows that the tensile strength reached 82.5% of that of the base metal, and the welding performance was good. During the tensile process of the joint, the easy fracture position was located at the intersection of material flow on the retreating side of the weld, and the fracture surface was at an angle of nearly 45° to the tensile direction.
- The Vickers hardness of the welded joint under this process parameter was tested, and the hardness of the upper part of the weld nugget area was higher than that of the middle and lower parts. The hardness of the advancing side of the weld nugget was slightly higher than that of the retreating side. The lowest Vickers hardness value of the joint appeared near the interface between the heat-affected zone and the heat-affected zone on both sides.
- After analyzing the weld performance, it was found that it could reach the aerospace application level, and the rocket roof was welded under this parameter. First of all, welding was carried out between the two melon petals of the top cover; melon petal welding was relatively difficult among the welding conditions, but a high-quality joint with good surface quality and no defects was obtained.

## Figures and Tables

**Table 1:** Dimension parameters of the tool.

Parameters	Value
Pin height	5.6 mm
Shoulder diameter	16.3 mm
Mix diameter of pin	8.15 mm
Tool tilt angle	2.5°
Plunge depth	0.25 mm
Concave angle of shoulder	10°
Taper angle of pin	15°

**Table 2:** Chemical composition of 2A14 aluminum alloys (wt %).

Cu	Si	Mn	Mg	Fe	Zn	Ti	Ni	Al
3.9–4.8	0.6–1.2	0.4–1.0	0.4–0.8	≤0.7	≤0.3	≤0.15	≤0.1	marginal

**Table 3:** Mechanical properties of 2A14 aluminum alloys.

Parameter	Value
Tensile strength *R*_m_/MPa	460
Percentage elongation after fracture *A* (%)	12
Vickers hardness (HV)	150–160

**Table 4:** Tensile test result of joint.

Sample	Tensile Strength Mpa	Percentage Elongation after Fracture *A* (%)
1	372.86	6.79
2	383.52	7.04
3	381.59	7.25
Average value	379.32	7.02
